# A Testis-Derived Hydrogel as an Efficient Feeder-Free Culture Platform to Promote Mouse Spermatogonial Stem Cell Proliferation and Differentiation

**DOI:** 10.3389/fcell.2020.00250

**Published:** 2020-05-19

**Authors:** Yan Yang, Qilian Lin, Chengxing Zhou, Quan Li, Ziyi Li, Zhen Cao, Jinlian Liang, Hanhao Li, Jiaxin Mei, Qihao Zhang, Qi Xiang, Wei Xue, Yadong Huang

**Affiliations:** ^1^Guangdong Provincial Key Laboratory of Bioengineering Medicine, Department of Cell Biology, Jinan University, Guangzhou, China; ^2^Department of Biomedical Engineering, Jinan University, Guangzhou, China; ^3^Department of Pharmacology, Jinan University, Guangzhou, China; ^4^Biopharmaceutical Research & Development Center of Jinan University, Guangzhou, China

**Keywords:** testicular extracellular matrix, hydrogel, spermatogonial stem cells, proliferation, differentiation

## Abstract

Fertility preservation and assisted reproductive medicine require effective culture systems for the successful proliferation and differentiation of spermatogonial stem cells (SSCs). Many SSC culture systems require the addition of feeder cells at each subculture, which is tedious and inefficient. Here, we prepared decellularized testicular matrix (DTM) from testicular tissue, which preserved essential structural proteins of testis. The DTM was then solubilized and induced to form a porous hydrogel scaffold with randomly oriented fibrillar structures that exhibited good cytocompatibility. The viability of SSCs inoculated onto DTM hydrogel scaffolds was significantly higher than those inoculated on Matrigel or laminin, and intracellular gene expression and DNA imprinting patterns were similar to that of native SSCs. Additionally, DTM promoted SSC differentiation into round spermatids. More importantly, the DTM hydrogel supported SSC proliferation and differentiation without requiring additional somatic cells. The DTM hydrogel scaffold culture system provided an alternative and simple method for culturing SSCs that eliminates potential variability and contamination caused by feeder cells. It might be a valuable tool for reproductive medicine.

## Introduction

Spermatogenesis is the proliferation and differentiation of spermatogonial stem cells (SSCs) called germ-line stem cells, within the seminiferous tubules of the testes, resulting in haploid, free-swimming spermatozoa. Maintenance of SSCs and induction of spermiogenesis *in vitro* will facilitate the study of reproductive biology, including assisted reproductive medicine and genetic modification (Stukenborg et al., 2009). SSCs are extremely rare (0.02–0.03%) in the testis (de Rooij and Russell, 2000); it is valuable to develop protocols for SSC propagation and differentiation *in vitro*.

SSCs located in the seminiferous tubules require complex endocrine and auto-/paracrine regulation as well as cellular interactions to proliferate and differentiate (de Rooij, 2009). SSC propagation requires growth factors, including glial cell line-derived neurotrophic factor (GDNF), secreted from Sertoli cells, basic fibroblast growth factor (bFGF), and epidermal growth factor (EGF) (Simon et al., 2007; Hofmann, 2008; Takashima et al., 2015). The widely adopted approach for SSC propagation was a two-dimensional culture system relying on cytokines of feeder cell layers. Mouse embryonic fibroblasts (MEFs) and Sertoli cells were the most frequently used feeder cells (Kanatsu-Shinohara et al., 2003; Nasiri et al., 2012; Song and Wilkinson, 2014). However, the preparation of subcultures for SSCs is tedious and variable. It is necessary to establish an efficient and practical method to propagate SSCs. Kanatsu-Shinohara et al. reported that laminin could maintain SSC activity when the medium was supplemented with GDNF, fetuin, and lipid-associated molecules (Kanatsu-Shinohara et al., 2011). However, the proliferative frequency of SSCs was lower (0.4–1.0%). Subsequently, Kinarm Ko et al. developed a Matrigel-based feeder-free culture system for long-term propagation of SSCs using the StemPro-34 SFM medium containing numerous factors for SSCs (Choi et al., 2014). Besides, it was reported that soft agar culture system (SACS) (Stukenborg et al., 2008) and collagen gel matrix (Khajavi et al., 2014) could mimic germ cell niche formation in the seminiferous tubules to permit mouse spermatogenesis *in vitro*. However, in these culture systems, somatic cells have a critical role in meiotic and postmeiotic differentiation of SSCs. Therefore, establishing an alternative culture condition that mimics testis niche is important for SSC proliferation and differentiation *in vitro*.

The extracellular matrix (ECM) consists of structural and functional molecules secreted by the resident cells, which provides biochemical and biomechanical signaling cues to influence the surrounding cells’ behavior dynamically (Agmon and Christman, 2016). It was used in tissue engineering. Thereinto, tissue-specific ECM scaffolds derived from homologous tissue may be more effective in producing desired cell phenotypes than non-homologous tissues (Zhang et al., 2009; Aamodt and Grainger, 2016; Spang and Christman, 2018). The ECM are available in sheets, powders, and hydrogels (Aamodt and Grainger, 2016; Saldin et al., 2017; Spang and Christman, 2018). The hydrogel is able to fill a different space with a homogeneous concentration. In the proper composition, the ECM hydrogel scaffolds can promote mesenchymal stem cell differentiation into a neural lineage (DeQuach et al., 2011), hepatocytes (Park et al., 2016). Recently, it has been reported that decellularized testicular matrix (DTM) may be a suitable material for the generation of the testicular organoids; however, the ability of DTM hydrogels to support SSC proliferation and differentiation in the absence of testicular cells remains unknown (Baert et al., 2015, 2017; Murdock et al., 2019; Rezaei Topraggaleh et al., 2019).

In this study, we produced a soluble DTM using an acid–pepsin solution, which self-assembled to form a hydrogel scaffold. The DTM-derived hydrogel scaffold could provide a microenvironment to support SSCs proliferation and differentiation *in vitro*.

## Materials and Methods

### Animals

Mice used in experiments were purchased from the Experimental Animal Center of Guangdong Province, China. Animals were maintained under a 12-h light/dark cycle and a controlled temperature (24 ± 2°C) with relative humidity (50–60%). The standard rodent diet and drinking water were freely accessible. All experiments were conducted according to the National Institute of Health guidelines for the care and use of animals and approved by the Institutional Animal Care and Use Committee of Jinan University.

### Decellularization of Testicular Tissue

Testes were obtained from male mice and decapsulated. Testicular fragments were decellularized as previously described (Baert et al., 2015). Briefly, tissues were rinsed in 1 × phosphate-buffered saline (PBS) to remove residual blood and subsequently stirred in 1% sodium dodecyl sulfate (SDS) in PBS solution for 18 h. Following the decellularization, the tissue was washed using PBS for 24 h, and PBS was renewed six times to remove the cellular fragment and remaining detergents. The decellularized testis-derived ECM, referred to as DTM, was lyophilized, and stored in −20°C until use.

### Assessment of Cellular Contents

Native and decellularized samples were fixed in 4% paraformaldehyde for 24 h, then embedded in paraffin, and serially sectioned at 5 μm thickness. The cell nuclei residues were determined by hematoxylin and eosin (H&E) staining or 4′,6-diamidino-2-phenylindole (DAPI) (Sigma, Poole, Dorset, United Kingdom) staining.

For quantitative analysis of the DNA content, DNA was extracted from native or DTM tissues using the HiPure Tissue DNA Mini Kit (Magen, Shanghai, China) according to the manufacturer’s instructions. The concentration of the total DNA was determined using a Nano Drop ND-2000 spectrophotometer (2000C, Thermo Fisher Scientific, United States) and normalized for sample weight.

### Immunohistochemistry

Retention of collagen I, collagen IV, fibronectin, and laminin in DTM was studied by immunohistochemistry. Sections were deparaffinized and rehydrated. Endogenous peroxidases were quenched using 0.3% H_2_O_2_. After blocking with 3% bovine serum albumin (BSA), sections were incubated with primary antibodies at 4°C overnight, followed by incubation using a horseradish peroxidase (HRP)-conjugated secondary antibody at 37°C for 1 h. The peroxidase activity was visualized with 3,3-diaminobenzidine (DAB) for 5 min, and the slides were counterstained with hematoxylin. Normal rabbit immunoglobulin G (IgG) was used for isotype controls. The following antibodies were used: rabbit anticollagen I (Affinity Biosciences, Changzhou, China), rabbit anticollagen IV (Affinity Biosciences, Changzhou, China), rabbit antifibronectin (Affinity Biosciences, Changzhou, China), and rabbit antilaminin (Affinity Biosciences, Changzhou, China).

### Preparation of DTM Hydrogel

DTM hydrogels were prepared according to previously described protocols (Pati et al., 2014). Briefly, lyophilized DTM powder was sterilized by e-beam irradiation at 22 kGy and stored at room temperature. Then, the sterilized DTM powder was digested in a solution of 0.5 M acetic with 10 mg of pepsin (Sigma, Poole, Dorset, United Kingdom) for 100 mg DTM and stirred at 4°C for 72 h to form a pregel solution under sterile condition. The solution was centrifuged to remove the particles. The pepsin–acetic DTM solution was neutralized to pH 7.0 with dropwise addition of cold 1 M sodium hydroxide (Sigma, Poole, Dorset, United Kingdom). To form the hydrogel, the neutralized pregel was incubated at 37°C for 60 min.

### Rheological Measurement

Rheological characterizations of DTM hydrogel were determined using a Physica MCR 301 rheometer (Anton Paar, Hertford, United Kingdom) as previously described (Paduano et al., 2017). Briefly, the pH of the DTM digest was neutralized to 7.0 and diluted to 2.5, 5, or 10 mg DTM/ml. The diluted pregel solutions at 4°C were placed between 50 mm parallel plates separated by a 0.2-mm measuring gap. The plates were precooled within a humidified chamber to 4°C and were then warmed to 37°C during the first 75 s of each measurement run. A 30-min time course experiment was performed, during which the samples were subjected to an oscillatory strain of 1% at a constant angular frequency of 1 rad/s (0.159 Hz) and rapidly increased temperature from 4 to 37°C to induce gelation as indicated by a sharp increase and plateauing of the storage modulus (*G*′), and the loss modulus (*G*″) (*n* = 3).

### Turbidity Gelation Kinetics Assay

Turbidimetric gelation kinetics was determined as previously described (Medberry et al., 2013). In brief, the neutralized DTM solution was diluted to 2.5, 5, and 10 mg/ml and placed into a 96-well plate (100 μl/well). The plate was placed in a preheated (37°C) plate reader, and the optical density (OD) was read at 405 nm every 2 min for 90 min. Data were collected using GraphPad prism and normalized using **Equation (1)**, where *A* is the absorbance at a given time, *A*_0_ is the lowest absorbance (time zero), and *A*_max_ is the maximum absorbance. According to the fitting curve, the following values can be obtained: the time required to 50% (*t*_1__/__2_) and 95% (*t*_95_) maximum absorbance. The lag phase (*t*_lag_) was defined as the point where a line representing the slope at log *t*_1__/__2_ intersects the turbidimetry baseline with 0% absorbance. The turbidimetric speed (*S*) was determined by calculating the slope of the curve at *t*_1__/__2_.

(1) $$\mathrm{Normalized}\,\mathrm{absorbance}\,\left(\mathrm{NA}\right)=\frac{A-A_{0}}{A\,m\,a\,x-A_{0}}$$

### Scanning Electron Microscopy

DTM hydrogels were frozen at −80°C and lyophilized overnight. Subsequently, the lyophilized hydrogel was dried in a critical point dryer and sputter coated with gold/palladium. Images were taken under a LEO 1530 scanning electron microscope. The average pore size of DTM was determined by the measurement of two perpendicular dimensions of at least 10 randomly selected pores using ImageJ software (ImageJ, United States).

### Spermatogonial Stem Cell Culture

Testicular tissue was obtained from 6-day-old male mice. To obtain SSC suspension from the tissue, a two-step enzymatic digestion protocol was applied (Guan et al., 2009). Briefly, decapsulated testes were treated with collagenase type IV (1 mg/ml) for 15 min at 37°C, followed by digestion in 0.25% trypsin and 1 mM ethylenediaminetetraacetic acid (EDTA) for 10 min at 37°C. The singly dissociated cells were incubated overnight in dish coated with gelation to removed somatic cells. Non-adherent and weakly adherent cells were collected and then labeled with CD326 (EpCAM) MicroBeads by Mini MACS Starting Kit (Miltenyi Biotec, Stevenage, United Kingdom) according to the manufacturer’s protocol. Cells were rinsed with PBS containing 0.5% BSA (Sigma, Poole, Dorset, United Kingdom), and the CD326-positive cells were collected. The CD326-positive cells were plated onto DTM hydrogel, Matrigel (10 mg/ml) hydrogel, or laminin (2 mg/ml) coated plates with SSC culture medium. The SSC medium was composed of Dulbecco’s modified Eagle’s medium (DMEM), 15% fetal bovine serum (FBS, Thermo Fisher Scientific, Waltham, MA, United States), 50 μM-mercaptoethanol (Thermo Fisher Scientific, Waltham, MA, United States), 1 × minimal essential medium (MEM) non-essential amino acids (Thermo Fisher Scientific, Waltham, MA, United States), and 10 ng/ml mouse GDNF (Peprotech, Rocky Hill, United States).

### Live/Dead Staining

Five hundred microliters of DTM hydrogel (2.5, 5, and 10 mg/ml), 10 mg/ml Matrigel growth factor reduced basement membrane matrix (Corning, New York, United States), and 2 mg/ml laminin (Sigma, Poole, Dorset, United Kingdom) were added to six-well plates and incubated at 37°C for 2 h to form scaffold. SSCs were seeded on laminin, DTM hydrogel, and Matrigel hydrogel for 7 days. The live/dead cells were visualized by Calcein-AM/PI Double Stain Kit (Yeasen, Shanghai, China). Samples were imaged under a microscope. Live (green) and dead (red) cells were counted using Image J software.

### Alkaline Phosphatase Reactivity

Cells were washed with PBS three times and fixed with 4% paraformaldehyde for 3 min. Alkaline phosphatase (ALP) (Leagene, Beijing, China) was added to the plate and incubated for 30 min and then washed with PBS three times. The samples were photographed under a microscope within 3 h.

### Reverse Transcription PCR

Reverse transcription PCR (RT-PCR) was performed to assess the expression of SSC-specific marker genes in colonies. RNA was extracted from the cell pellet by Trizol reagent (Sigma, Poole, Dorset, United Kingdom). Reverse transcription was performed using Prime Script^TM^ RT Master Mix (Takara, Dalian, China) following manufacturer’s instructions. The primers are listed in Supplementary Table S1.

### Flow Cytometric Analysis

To determine the expression of SSC-specific surface markers, SSCs were resuspended in cold PBS supplemented with 0.5% FBS at density of 1 × 10^6^ cells/ml and stained with primary antibodies, CD326 (EpCAM), CD49f (Intergrin α6), CD29 (Intergrin β1), and CD117 (c-kit) (all from EBioscience, Thermo Fisher Scientific, Waltham, MA, United States), at 4°C for 30 min. Rabbit IgG1-PE was used as isotype control. Flow cytometry was performed using CellQuest software (BD Biosciences).

### Genomic DNA Methylation Analysis

Genomic DNA of SSCs was isolated using the HiPure Tissue DNA Mini Kit (Magen, Shanghai, China) according to the manufacturer’s protocol. The methylation levels of the *H19* and *Igf2r* gene of genomic DNA were determined by bisulfite sequencing PCR. The primers are shown in Supplementary Table S1.

### Spermatogonial Stem Cell Transplantation

Transplantation was performed as previously described (Ko et al., 2009). Briefly, 4-week-old male mice were treated with 25 mg/kg busulfan by intraperitoneal injection. After 1 month, these mice were used as SSC recipients. SSCs cultured on DTM were collected and labeled with carboxyfluorescein diacetate succinimidyl ester (CFDA-SE) following manufacturer’s instructions. Then, the single-cell suspensions (approximately 1 × 10^5^ cells in 10 μl of PBS) were injected into the seminiferous tubules of recipient mice through the efferent ducts using a micropipette (40–50 μm diameter tips). The mice were euthanized 1 month later, and testes were collected and examined.

### Spermatogonial Stem Cell Differentiation

SSCs were plated on laminin, Matrigel, and DTM hydrogel scaffolds with SSC differentiation medium composed of DMEM, 10% FBS, minimal essential medium (MEM) non-essential amino acids (Thermo Fisher Scientific, Waltham, MA, United States), 10 μM testosterone (Cayman, Michigan, United States), 100 ng/ml follicle stimulating hormone (FSH) (ProSpec, Rocky Hill, United States), and 100 ng/ml retinoic acid (RA) (Sigma, Poole, Dorset, United Kingdom) at 34°C under 5% CO_2_. The culture medium was changed every 2 days.

### Quantitative Real-Time PCR

RNA was extracted from the cell pellet by Trizol reagent (Sigma, Poole, Dorset, United Kingdom). Reverse transcription was performed with the complementary DNA (cDNA) synthesis kit (TaKaRa Taq^TM^ Version 2.0 plus dye). Gene expression was analyzed quantitatively using the ChamQ SYBR^®^ qPCR Master Mix (Vazyme, Nanjing, China). The PCR data were recorded by Bio-Rad CFX Manager Software (version 2.0). Gene expression data were normalized to the *Gapdh* as housekeeping gene. The primers are listed in Supplementary Table S2.

### Immunofluorescence

Cells were fixed in 4% paraformaldehyde for 30 min and washed three times in PBS. The cells were treated in 0.2% Triton-X for 10 min. Non-specific adhesion sites were blocked with 3% BSA (Sigma, Poole, Dorset, United Kingdom) for 30 min at room temperature. The primary and secondary antibodies were diluted in a solution of PBS containing 3% BSA, 1% horse serum, and 0.1% Triton X-100. Cells were incubated with primary antibodies Acrosin (Santa, CA, United States) overnight at 4°C, followed by incubation with secondary antibodies for 2 h at room temperature. Nuclei were stained with DAPI (Thermo Fisher Scientific, Waltham, MA, United States). Stained samples were visualized, and images were captured using a LSM710 confocal microscope (Zeiss) and analyzed by the Image J software.

### DNA Content Analysis

Flow cytometry was performed to measure the DNA content of SSCs. In brief, cells were washed twice in PBS and fixed in cold 70% ethanol for 4 h. Cells were then stained with propidium iodide at 2–8°C for 10 min and analyzed within 3 h by FACSCalibur system (BD Bioscience).

### Statistical Analyses

All experiments were repeated at least three times, and data were expressed as the mean ± one standard deviation around the mean (SD). Statistical analyses were performed by Prism software (GraphPad Software, San Diego, CA, United States). Statistical analyses were performed with an unpaired Student’s *t* test or one-way ANOVA for more than two groups. A two-tailed value of *p* < 0.05 was considered statistically significant.

## Results

### Macroscopic Appearance of Decellularized Testicular Matrix

**FIGURE 1:**
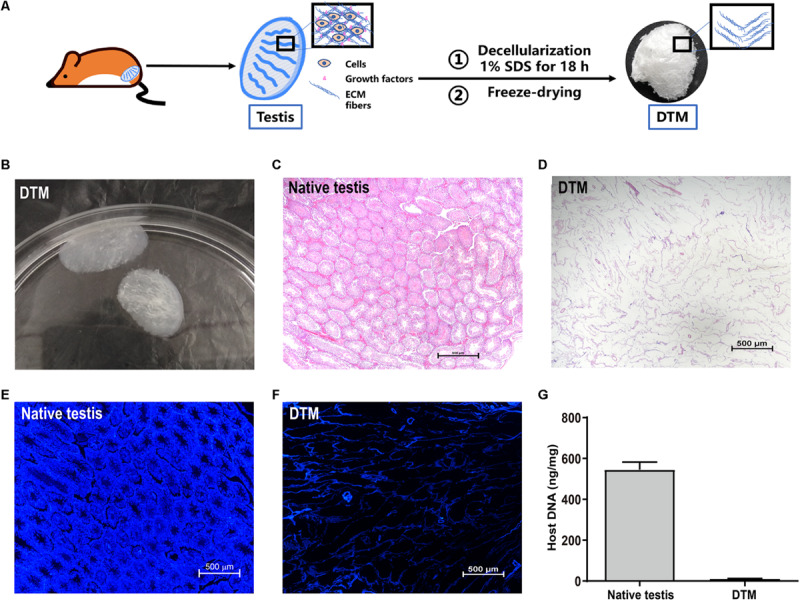
Characterization of native testes and decellularized testicular matrix (DTM). **(A)** Schematic illustration showing the process of DTM generation. **(B)** Macroscopic appearance of DTM. **(C,D)** H&E staining of testis section before decellularization and DTM. Scale bar, 500 μm. **(E,F)** 4′,6-Diamidino-2-phenylindole (DAPI) staining for residual DNA content in testis section before decellularization and DTM. Scale bar, 500 μm. **(G)** DNA quantification of native testis and DTM.

**FIGURE 2:**
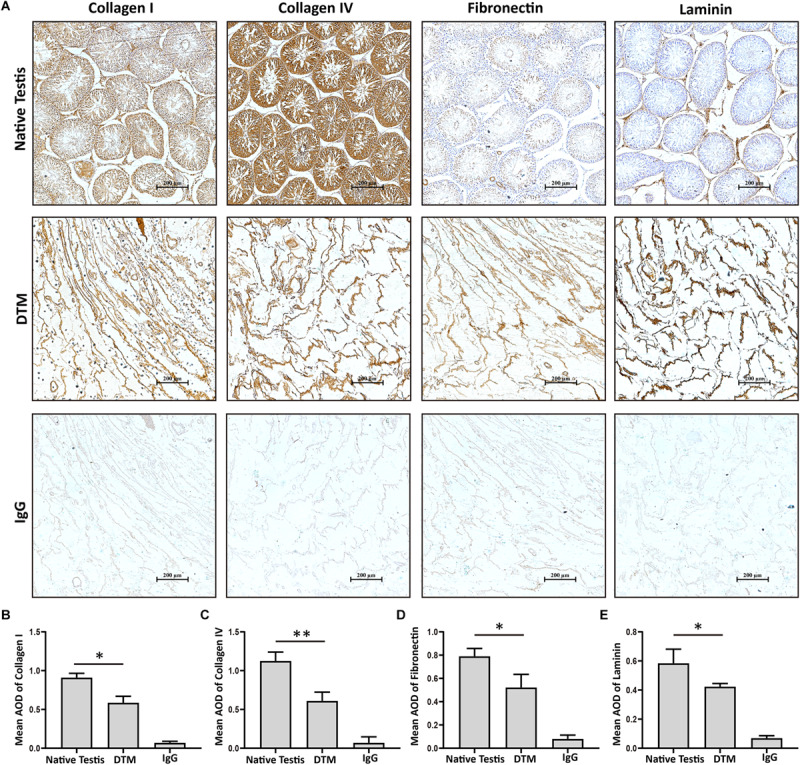
Immunohistochemical staining of native testis and decellularized testicular matrix (DTM). **(A)** Immunohistochemical validation of the essential extracellular matrix proteins of the native testis (laminin, collagen type I, collagen type IV, and fibronectin) in the DTM. Scale bar, 200 μm. **(B–E)** Relative quantity of collagen I, collagen IV, fibronectin, and laminin. ImageJ software was used to determine the average optical density (AOD). Data were shown as mean ± SD, *n* = 8. Differences were considered statistically significant at *p < 0.05, **p < 0.01.

Testes were collected and the tunica albuginea was removed. The exposed testicular tissues were treated with 1% SDS to generate a DTM (Figures 1A,B). H&E staining displayed the absence of cells and cell debris in the matrix after decellularization (Figures 1C,D). DAPI staining also confirmed the absence of residual nuclei in the DTM when compared with the native testicular tissue (Figures 1E,F). The residual DNA content in the DTM, 11.37 ± 0.84 ng/mg, was significantly lower than that of the native testes tissue (545.20 ± 37.21 ng/mg) (Figure 1G), indicating that 97.91% of DNA was removed from the tissue. The minimal residual cell debris and DNA indicated that the testicular tissue was successfully decellularized. We then evaluated the retention of structural components in DTM following decellularization. Immunohistochemical analysis revealed that the expression of laminin, fibronectin, collagen type I, and collagen type IV were significantly reduced after decellularization. However, the presence of laminin, fibronectin, collagen type I, and collagen type IV in DTM indicated that the essential components of testicular ECM were preserved in DTM after decellularization (Figure 2).

### Characterization of Decellularized Testicular Matrix Hydrogel

**FIGURE 3:**
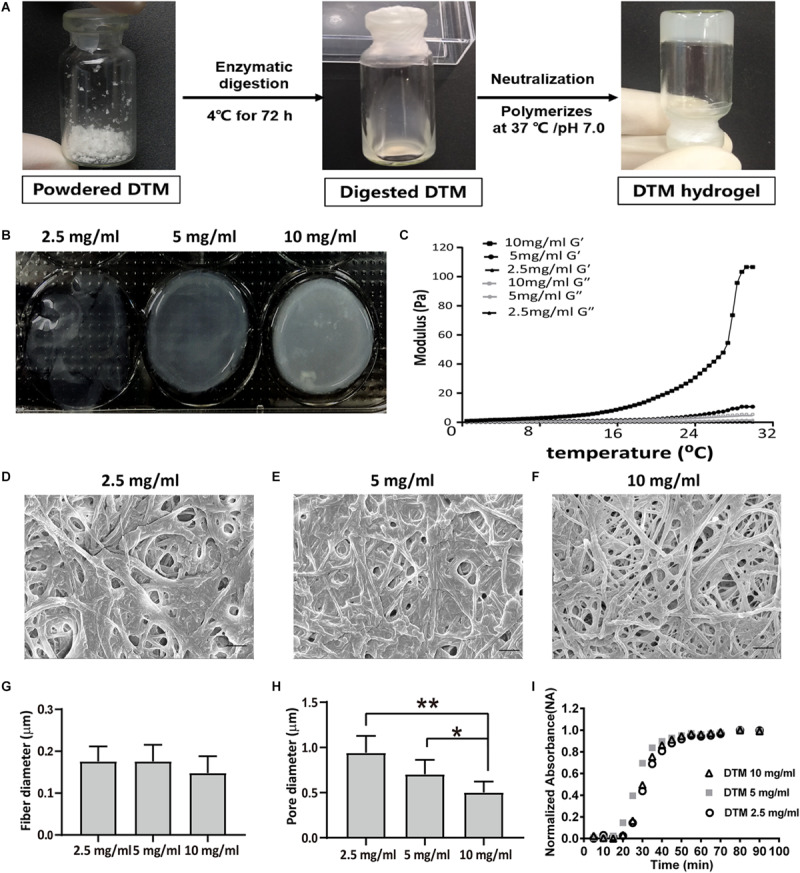
Characterization of decellularized testicular matrix (DTM) hydrogel with DTM concentrations of 2.5, 5, and 10 mg/ml. **(A)** Schematic illustration showing the production of DTM hydrogel. **(B)** Macroscopic view of pH-neutralized DTM digests injected into 1.38 cm diameter rings at 37°C for 1 h. **(C)** Rheological characterization of DTM hydrogels. The gelation kinetics were determined by monitoring changes in the storage modulus (*G*′) and loss modulus (*G*″) after inducing gelation. Data represent the means for three independent experiments. **(D–F)** Scanning electron microscopy (SEM) images of DTM derived hydrogels formed at DTM concentrations of 2.5, 5, and 10 mg/ml. The scale bars represent 1 μm. **(G,H)** Fiber diameter and pore size of DTM hydrogels at concentrations of 2.5, 5, and 10 mg/ml. SEM images were analyzed using an automated fiber-tracking algorithm to determine the average fiber diameter and pore size of each concentration. Data were shown as mean ± SD, *n* = 6. Differences were considered statistically significant at *p < 0.05, **p < 0.01. **(I)** Turbidimetric gelation kinetics of DTM hydrogel at DTM concentrations of 2.5, 5, and 10 mg/ml. After DTM pepsin digests were pH neutralized, DTM were inoculated into a 96-well plate at 37°C to induce gelation. The absorbance at 405 nm was measured at 5-min intervals and normalized between 0 (the initial absorbance) and 1 (the maximum absorbance).

The lyophilized DTM was digested in pepsin solution; then, the DTM solution was thermally induced to form DTM hydrogels (Figure 3A). The hydrogels with a low DTM concentration (2.5 mg/ml) were extremely delicate. Hydrogels with DTM concentration of 5 and 10 mg/ml had a rigid structure with defined edges (Figure 3B). The rheological characteristics of the DTM hydrogels were determined using a parallel plate rheometer. After incubating the pregels at 37°C, their behavior was like that of a cross-linked gel and exhibited greater storage modulus (*G*′) than loss modulus (*G*″). Results displayed that pregel with a DTM concentration of 10 mg/ml had the highest storage modulus exhibiting solid-like behavior (Figure 3C).

The fiber network topology revealed that DTM hydrogels possessed a randomly oriented fibrillar structure (Figures 3D–F), and average fiber diameter did not vary with DTM concentration (Figure 3G). The pore size of the hydrogels with a DTM concentration of 10 mg/ml was 0.5 μm, which was significantly smaller than the pore size of hydrogels at DTM concentration of 5 mg/ml (0.70 μm) and 2.5 mg/ml (0.94 μm) (Figure 3H).

The turbidimetric gelation kinetic curves for the hydrogels were sigmoidal at different DTM concentrations. Hydrogels formed after a lag period. The half time of gelation (*t*_1__/__2_) for the hydrogels was not dependent on DTM concentration: 2.5 mg/ml DTM, 30 min; 5 mg/ml DTM, 27 min; and 10 mg/ml DTM, 30 min. Similarly, no significant difference was found in either time of 95% gelation (*t*_95_) or lag time (*t*_lag_) at various DTM concentrations (Figure 3I).

### Decellularized Testicular Matrix Hydrogel Enhanced SSC Survival

**FIGURE 4:**
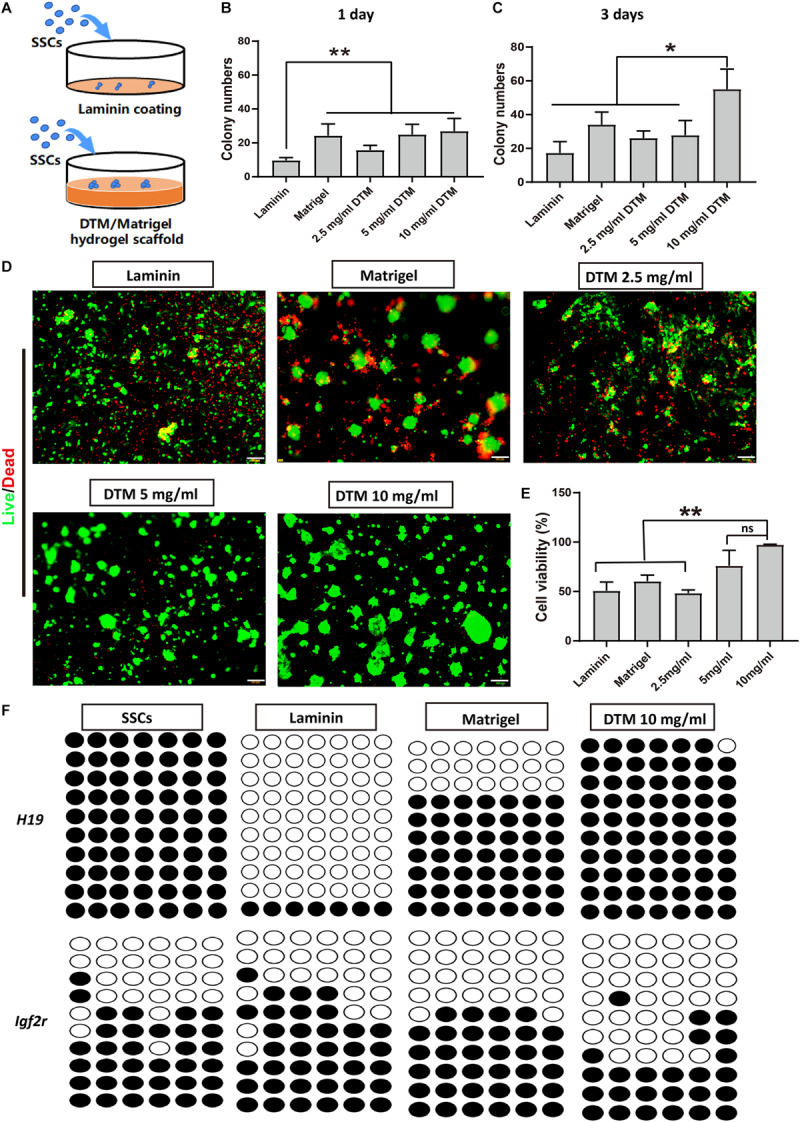
Decellularized testicular matrix (DTM) hydrogel enhances spermatogonial stem cell (SSC) survival. **(A)** Schematic diagram of the experimental design. **(B,C)** The number of SSC colonies were analyzed at days 1 and 3. SSC cultured on the DTM hydrogels (2.5, 5, and 10 mg/ml) scaffold, Matrigel scaffold, and laminin-coated dish with SSC medium treatment. The number of colonies were measured by ImageJ software at a sample size of 100–500 μm^2^ at the end of the first and third day. **(D)** Live/dead assays revealing the morphologies of SSC cultured on the DTM hydrogels with DTM concentrations of 2.5, 5, and 10 mg/ml, Matrigel, and laminin-coated dishes after 7 days. Green indicates live cells, and red indicates dead cells. Scale bar, 100 μm. **(E)** Cell survival rates of SSC were analyzed at day 7. Data are shown as mean ± SD, *n* = 6. Differences were considered statistically significant at **p < 0.01. ns, no significant. **(F)** DNA methylation pattern of *H19* and *Igf2r* gene in SSCs. The DNA methylation status of paternally imprinted (*H19*) and maternally imprinted (*Igf2r*) genes in SSCs cultured on 10 mg/ml DTM hydrogels, Matrigel scaffold, and laminin-coated dish at day 7 were analyzed by bisulfite sequencing. Black circles represent methylated CpG sites, and white circles represent unmethylated CpG sites. CpG, 5′-C-phosphate-G-3′.

The effect of DTM hydrogel on the adherence and proliferation of SSC was investigated. SSCs were plated on DTM hydrogel (2.5, 5, and 10 mg/ml) scaffold, Matrigel scaffold, and laminin-coated dish for 7 days (Figure 4A). At the first day, SSCs spontaneously formed aggregates on all concentrations of DTM hydrogels and Matrigel scaffold but not on laminin-coated dish (Figure 4B). The number of SSC colonies formed on the 10 mg/ml DTM hydrogel was significantly higher than the other groups after the third day (Figure 4C).

After 7 days, calcein-AM and propidium iodide double staining revealed that most of the SSCs on the 10 mg/ml DTM hydrogels displayed high viability, indicated by green dots, and expanded on the surface of the scaffold. Additionally, there was very few dead cells on 10 mg/ml DTM hydrogel, as indicated by red dots (Figure 4D). The percentage of viable SSC, 96.19 ± 3.11%, was significantly higher on 10 mg/ml DTM hydrogels than on laminin (50.71 ± 7.21%) or Matrigel (60.31 ± 5.06%) (Figure 4E).

Besides, the epigenetic stability of SSCs cultured on 10 mg/ml DTM hydrogels, Matrigel, and laminin was analyzed by bisulfite sequencing to determine the genomic imprinting pattern of the SSCs. Differentially methylated regions (DMRs) of the paternally imprinted *H19* gene and maternally imprinted *Igf2r* gene were examined. The level of *H19* methylation and *Igf2r* demethylation in SSCs seeded on DTM hydrogels was closer to native SSCs when compared with laminin and Matrigel. SSCs on Matrigel and laminin had decreased *H19* methylation, and the hypomethylation level of *Igfr2* was elevated (Figure 4F). Taken together, these results indicated that 10 mg/ml DTM hydrogel scaffolds provided a better environment to support SSC survival and maintain the native DNA methylation patterns of SSC; therefore, this concentration was used for further study.

### Decellularized Testicular Matrix Maintains Stemness of SSCs

**FIGURE 5:**
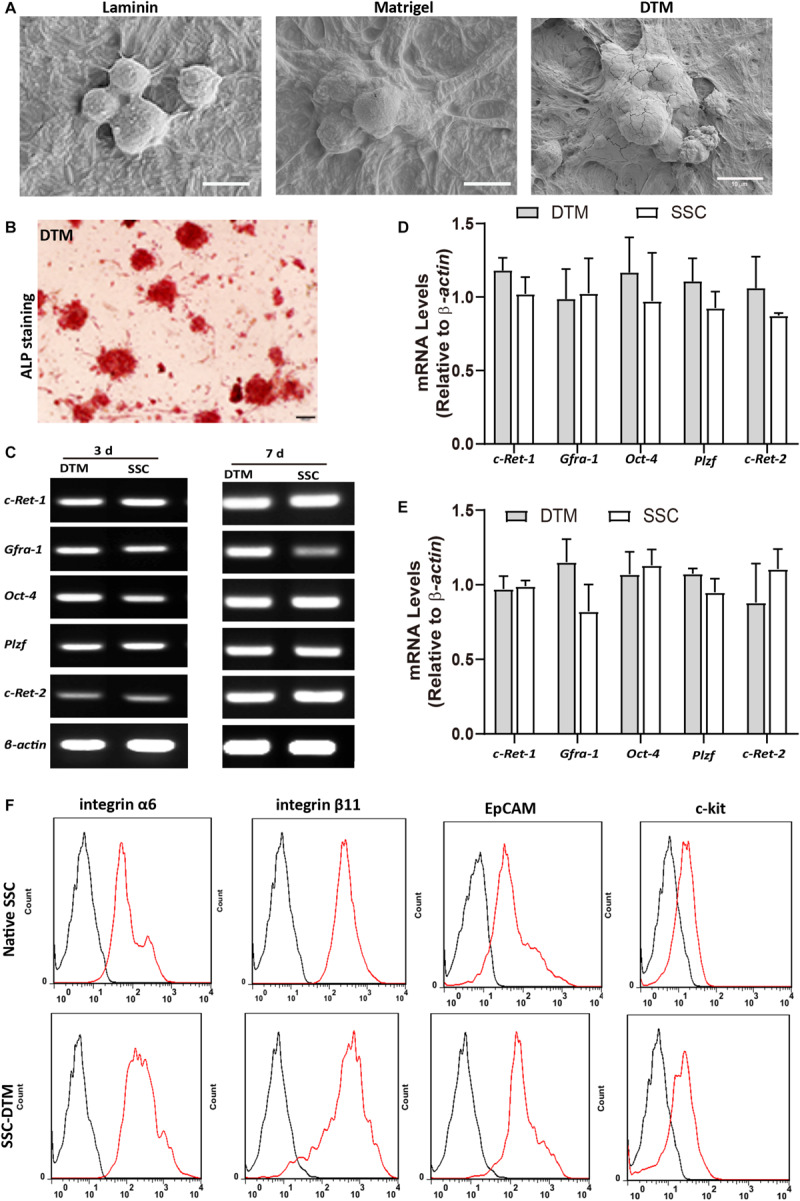
Decellularized testicular matrix (DTM) hydrogel maintains stemness of spermatogonial stem cells (SSCs). **(A)** Scanning electron microscopy (SEM) image of SSCs seeded on 10 mg/ml DTM hydrogel at day 7. Scale bar, 10 μm. **(B)** Alkaline phosphatase activity of SSCs seeded onto 10 mg/ml DTM hydrogels at day 7. Scale bar, 100 μm. **(C)** Reverse transcription PCR (RT-PCR0 analysis of SSC-specific gene expression after 3 and 7 days of culture on 10 mg/ml DTM hydrogel scaffold. **(D,E)** The amount of relative expression was normalized to that of β-actin. **(F)** Fluorescence-activated cell sorting (FACS) analysis of the expression of SSC surface proteins after 7 days of culture on 10 mg/ml DTM hydrogel scaffold. The red histograms represent the cell count for the specific antibody, and the back histograms represent the fluorescence of the negative control.

SSCs cultured on 10 mg/ml DTM hydrogels for 7 days displayed good adherence and colony formation (Figure 5A). Alkaline phosphatase activity was positive in the colonies formed on the DTM hydrogels (Figure 5B). SSCs cultured on DTM hydrogels expressed the SSC-specific gene markers (*Gfra1*, *c-Ret-1*, *c-Ret-2*, *Plzf*, and *Oct4*) (Figure 5C). There was no significant difference between SSC cultured on DTM and native SSC in expression (Figures 5D,E). SSC-specific surface proteins, integrin α6 (CD49f), integrin β6, and EpCAM (CD326) were positive in SSCs seeded on DTM hydrogel, while c-kit was negative (Figure 5F). However, the percentage of CD49f, integrin β6, and CD326 significantly decreased when SSC was seeded on laminin or Matrigel (Supplementary Figure S1). Therefore, the levels of genes and surface proteins of SSCs cultured on DTM hydrogels were similar to those of the native SSCs.

**FIGURE 6:**
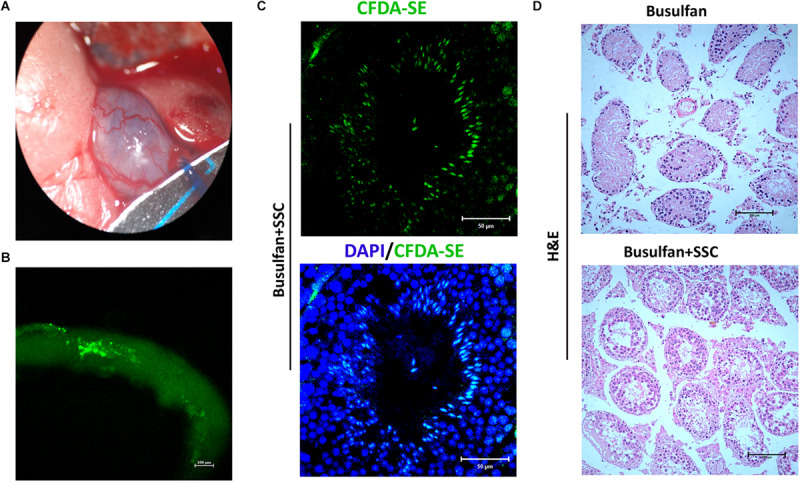
Spermatogonial stem cell (SSC) transplants into testicular tubules. **(A)** Schematic illustration of SSC transplantation. Approximately 5 × 10^5^ SSCs cultured on decellularized testicular matrix (DTM) hydrogels were micropipetted into the seminiferous tubules of mice 1 month after busulfan treatment (40 mg/kg). **(B)** Image of the recipient testicular tubule with fluorescence staining 35 days after SSC transplantation. **(C)** The testes were sectioned and stained with 4′,6-diamidino-2-phenylindole (DAPI) (blue) and examined under a fluorescent microscopy. Scale bars, 50 μm. **(D)** H&E staining of non-transplanted and transplanted recipient testis. The scale bars represent 200 μm.

A spermatogonial transplantation assay was performed to determine the stem cell activity of SSCs cultured on DTM hydrogel. The SSCs were labeled with CFDA-SE, a fluorescent green dye, and transplanted into the seminiferous tubules of busulfan-treated adult mice through their efferent ducts (Figure 6A). One month after transplantation, the mice were euthanized, and their testes were removed and examined for expression of CFDA-SE. SSCs were observed along the rim of the tubules, indicating that they had settled into their designated niche, the basal compartment of the seminiferous tubules (Figure 6B). Histological analyses displayed that some meiotic and postmeiotic cells labeled with CFDA-SE were observed in the tubules of SSC-transplanted mice. Only spermatogonia were present in the control (Figures 6C,D). These results suggested that the DTM hydrogel scaffold could maintain stemness of SSCs *in vitro*, and these SSCs could colonize into testicular tubules and differentiate after transplantation.

### DTM Hydrogel Promoted SSC Differentiation Into Round Spermatids

**FIGURE 7:**
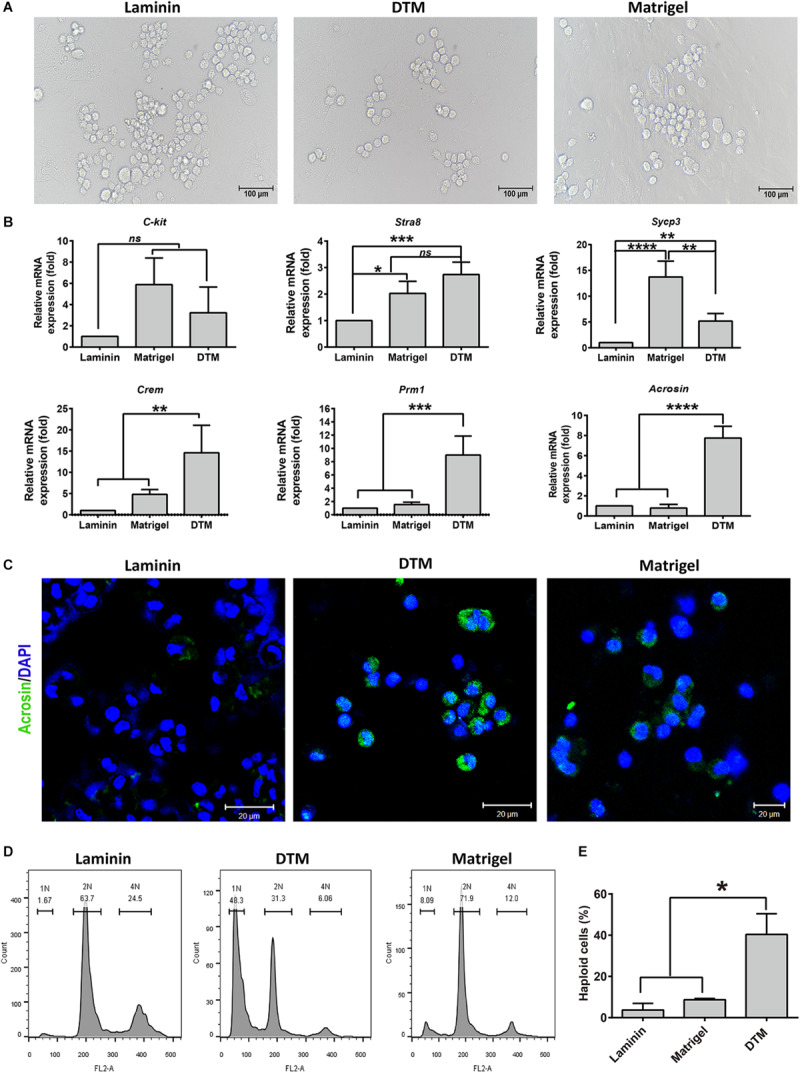
Assessment of the spermatogonial stem cell (SSC) differentiation in decellularized testicular matrix (DTM) hydrogel culture system. **(A)** Images of cell differentiation under a phase contrast microscope. Scale bar, 100 μm. **(B)** Real-time PCR analysis of the gene expression involved in spermatogonial differentiation or meiosis. **(C)** Immunofluorescence showing the expression of Acrosin in the differentiated cells from SSCs. The scale bars represent 20 μm. **(D)** Flow cytometry showing DNA content in the differentiated cells from SSCs. **(E)** The percentage of haploid cells. SSCs cultured on DTM hydrogel, Matrigel scaffold, and laminin-coated dish with SSC differentiation medium treatment for 7 days. All quantitative data were obtained from three independent experiments and are presented as mean ± SD; differences were considered statistically significant at ***p < 0.001, **p < 0.01, and *p < 0.05.

The influence of 10 mg/ml DTM hydrogels on SSC differentiation was evaluated by the addition of differentiation medium containing DMEM supplemented with FBS, testosterone, FSH, and RA for 7 days. Matrigel scaffold and laminin-coated dishes were used as controls (Figure 7A). The genes involved in spermatogonial differentiation or meiosis, *c-Kit*, *Stra8*, *Sycp3*, *Crem*, *Prm1*, and *Acrosin* were detected by quantitative RT-PCR (qRT-PCR). DTM hydrogel and Matrigel significantly promoted cells to express *c-Kit*, *Stra8*, and *Sycp3*. Meanwhile, the expression level of postmeiotic genes (*Crem*, *Prm1*, and *Acrosin*) in cells cultured on DTM hydrogel was greater than that on Matrigel and laminin (Figure 7B). Immunofluorescence revealed that more cells expressed acrosin, a protein of spermatids, in DTM compared to the Matrigel and laminin (Figure 7C and Supplementary Figure S2).

Furthermore, ploidy of SSCs on DTM, Matrigel, and laminin was assessed by flow cytometry. Round or elongated spermatids should be haploid (1N) as the result of two consecutive meiotic divisions; spermatogonia, secondary spermatocytes; somatic cells should be diploid (2N); and primary spermatocytes should be tetraploid (4N). On an average, the percentage of haploid cells was 40.4% in DTM greater than that of Matrigel. The cells cultured on laminin largely consisted of 2N and 4N subpopulations with a negligible amount of 1N cells, indicating cell arrest at the 2N or 4N stage (Figures 7D,E). Collectively, DTM hydrogel scaffold induced SSC differentiation into round spermatids without requiring the additional somatic cells. DTM hydrogel scaffolds may support SSC differentiation better than Matrigel and laminin.

## Discussion

*In vivo*, the functionality of SSCs is maintained by the testicular microenvironment (Dores et al., 2012). Tissue-specific ECM mimics the microenvironment of native tissue, which is important in stem cell proliferation and differentiation. It has been reported that ECMs were potential substrates for expansion of retinal progenitor cells (Kundu et al., 2016) and mesenchymal stem cells (Lin et al., 2012; Ng et al., 2014). To determine whether the testicular ECM supported SSCs’ viability and proliferation effectively, we prepared DTM from the testicular tissue. After decellularization, 97.91% of cellular materials (DNA, cytosolic proteins) were removed. An immunohistochemistry analysis revealed that the essential proteins (collagen I, collagen IV, fibronectin, and laminin) that confer the structural and functional properties of the testis were preserved.

Since three-dimensional hydrogel scaffolds have a complex structure, they absorb and retain large volumes of water and exhibit plasticity similar to that of the microstructure of native extracellular matrix (Tibbitt and Anseth, 2009; Geckil et al., 2010), which greatly influence cell behavior (Lee et al., 2017; Romero-Lopez et al., 2017). The lyophilized DTM was solubilized by pepsin digestion and subsequently thermal induced to form porosity hydrogel scaffold. The hydrogel scaffolds possessed a randomly oriented fibrillar structure, and the pore size decreased with increased DTM concentrations, and exhibited sigmoidal gelation kinetics, which consistent with a nucleation and growth mechanism (Zhu et al., 2018). Previous studies (Kanatsu-Shinohara et al., 2011; Choi et al., 2014) have demonstrated that Matrigel and laminin supported SSC proliferation, but in this case, special SSC culture medium components, such as StemPro supplement, GDNF, fibroblast growth factor 2 (FGF2), stromal cell-derived factor-1 (SDF-1), EGF, and fetuin, were required. The medium was relatively complex, expensive, and time consuming to prepare. In our study, when SSCs were cultured on DTM hydrogel, it was able to attach and proliferate without DNA imprinting pattern changing in the absence of StemPro supplement and fetuin. However, laminin and Matrigel could not maintain the DNA imprinting pattern of SSC without StemPro supplement and fetuin. Besides, SSC propagated on DTM hydrogels displayed significantly higher cell viability and number of adherent colonies than that on Matrigel and laminin. The purity of SSC increased when the culture time extended. As a result, the gene expression of *Gfra-1* increased at day 7. These results indicated that DTM contained some factors that could maintain SSC activity and gene expression. The reason might be that pepsin cleaves the peptide bonds of the collagen triple helix structure to unravel collagen fibril aggregates (Miller, 1972) and produces a broad variety of bioactive peptides (Wu et al., 2015), which might play major roles in regulating cell proliferation, gene expression, and differentiation *in vitro*.

Regarding the differentiation of SSC, collagen gel matrixes (Lee et al., 2006, 2007), 3D soft agar culture system (SACS) (Stukenborg et al., 2008, 2009; Mahmoud, 2012), and 3D methylcellulose culture system (MCS) (Stukenborg et al., 2008; Khajavi et al., 2014; Huleihel et al., 2015) could induce murine male premeiotic germ cell differentiation into postmeiotic germ cell. However, these procedures depended on Sertoli cells. In our study, the hydrogel of DTM maintained SSCs’ properties and promoted SSC differentiation into round spermatids without somatic cells, and the number of haploid cells in the DTM hydrogels was significantly higher than that in Matrigel and laminin. This result was in agreement with previous observations showing that native ECM hydrogel scaffolds directed stem cells to differentiate into a specific lineage, including osteogenic cells (Pati et al., 2015) and adipose stem cells (Yang et al., 2013). Native ECM hydrogel could provide a favorable environment for stem differentiation (Hoshiba et al., 2016; Saldin et al., 2017; Spang and Christman, 2018). Although we did not have a detailed description on the composition of the DTM, survival and meiosis occurrence in the DTM hydrogel scaffold were observed. The DTM hydrogel might support some bioactive substances to promote SSC differentiation into round spermatids, while Matrigel and laminin lack the complex organic components of testicular ECM. Therefore, the composition of the DTM deserves careful study in the future.

In summary, we found that hydrogel scaffolds containing 10 mg/ml DTM mimicked the testicular microenvironment, providing an alternative feeder-free culture system for SSC proliferation and differentiation. The hydrogel scaffold culture systems could provide us an efficient model to study the survival, proliferation, and meiosis of SSCs *in vitro*. Compared with the feeder culture system, the DTM hydrogel eliminates potential variability and contamination caused by feeder cells, and the process of testis-derived hydrogel was easier to standardize. This would contribute important knowledge to the fields of reproductive and cellular biology.

## Conclusion

DTM was prepared from testicular tissues and induced to form porosity hydrogel scaffolds that possessed a randomly oriented fibrillar structure. Hydrogel scaffold containing 10 mg/ml DTM maintained the properties of SSCs at the molecular and cellular levels and promoted SSCs differentiating into round spermatids in the absence of somatic cells. The DTM hydrogel scaffold culture system is a simple and efficient feeder-free culture system and may provide a better environment to support SSC proliferation and differentiation. It might be a valuable tool for reproductive medicine.

## Data Availability Statement

The data that support the findings of this study are available from the corresponding author upon reasonable request.

## Ethics Statement

The animal study was reviewed and approved by Institutional Animal Care and Use Committee of Jinan University. Written informed consent was obtained from the owners for the participation of their animals in this study.

## Author Contributions

YY, QiL, and CZ conducted the experiments. QuL, ZL, CZ, JL, HL, and JM analyzed the data. QZ, QX, and WX interpreted the data. YY and YH designed the research and wrote the manuscript. All authors reviewed, edited, and approved the manuscript.

## Conflict of Interest

The authors declare that the research was conducted in the absence of any commercial or financial relationships that could be construed as a potential conflict of interest.
