# Supplementary material for: A Testis-Derived Hydrogel as an Efficient Feeder-Free Culture Platform to Promote Mouse Spermatogonial Stem Cell Proliferation and Differentiation
Source: Front Cell Dev Biol. 2020 May 19;8:250. doi: 10.3389/fcell.2020.00250 (PMC7248195; doi:10.3389/fcell.2020.00250)
Supplement: Supplementary file 1 [file Data_Sheet_1.docx]

**Supplemental Tables and figures**

**Table S1. Primers for the bisulfite sequencing PCR and RT-PCR**

| **Gene** | **Sense primer** | **Antisense primer** |
| --- | --- | --- |
| *H19* | TAAGGAGATTATGTTTTATTTTTGG | CACAACATTACCATTTATAAATTCC |
| *Igf2r* | GAGTGTGGTATTTTTATGTATAGTTAGG | TCRAAAAATTCTATAATCAAAACCAAC |
| *C-ret-1* | CAGCGGTGTCTCCATCCAGTA | CCCCTCGCTCGTGTCCTCCAA |
| *C-ret-2* | GGACCACCCAGATGAAAGGG | CGGGTCTGACATGCCATAGA |
| *Gfrα-1* | GGCCTACTCGGGACTGATTGG | GGGAGGAGCAGCCATTGATTT |
| *Plzf* | GAGACACACAGACAGACCCATACT | CACACATAACACAGGTAGAGGTACG |
| *Oct-4* | AGAAGGAGCTAGAACAGTTTGC | CGGTTACAGAACCATACTCG |
| *β-actin* | CAGCCTTCCTTCTTGGGTAT | TGGCATAGAGGTCTTTACGG |

**Table S2. Primers for real-time PCR**

| **Gene** | **Sense primer** | **Antisense primer** |
| --- | --- | --- |
| *Stra8* | GTTTGCCACCTGCAACTCAG | GGGCTCTGGTTCCTGGTTTA |
| *C-kit* | CAGGAGCAGAGCAAAGGTGTA | TGGGCCTGGATTTGCTCTT |
| *Sycp3* | GGGGCCGGACTGTATTTACT | CTTCCACCAGGCACCATCTT |
| *Crem* | TTTTTGAACTGCTGGCGACC | TGGACTTGGCCCTCTACACT |
| *Prm* | GGAGGAGGCGAAGATGCT | TTCATCGGACGGTGGCATTT |
| *Acrosin* | TAACACCACGTTGGTCCCTG | GCTCCGAAAACCAGTCTCCA |
| *β-actin* | CAGCCTTCCTTCTTGGGTAT | TGGCATAGAGGTCTTTACGG |


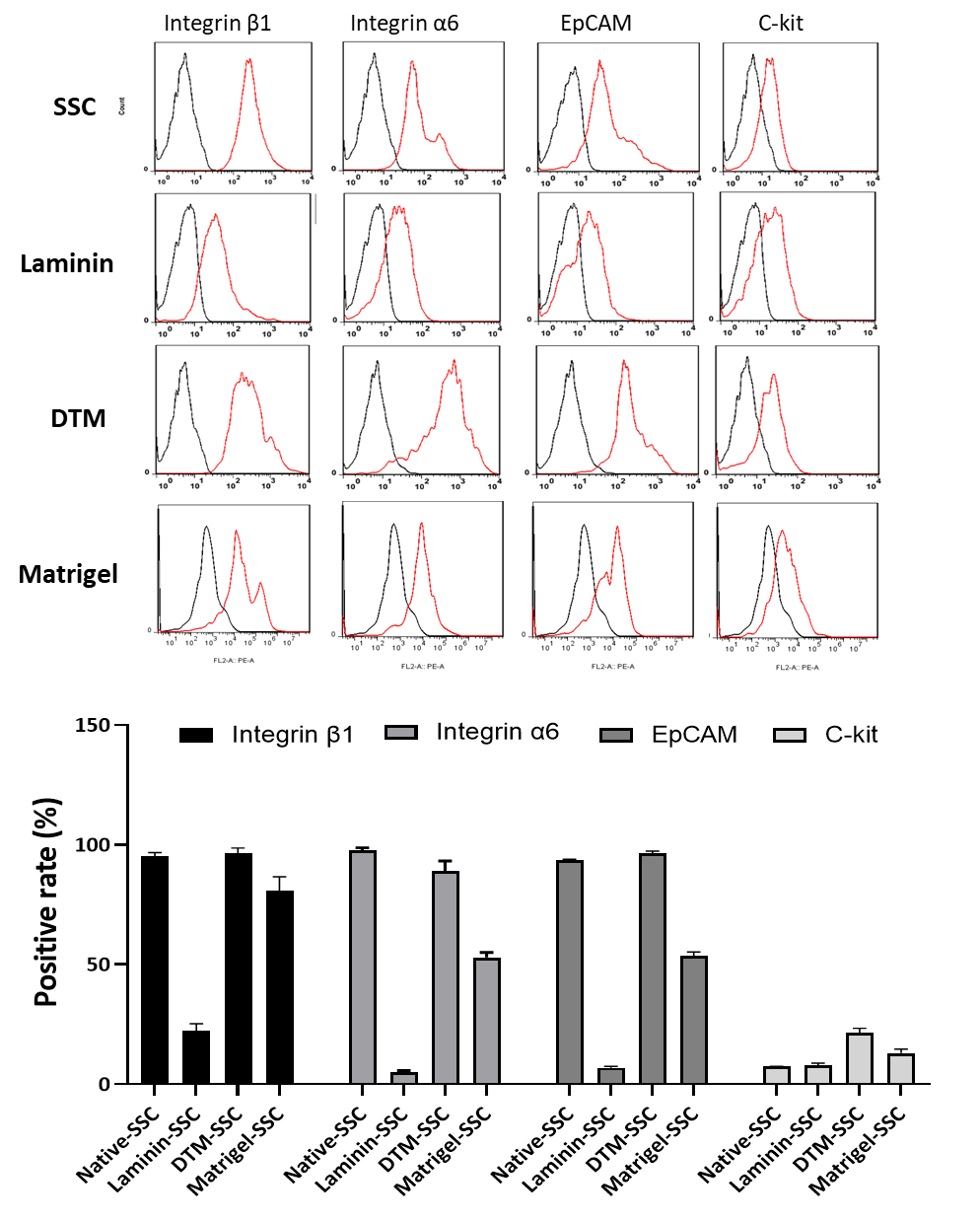


**Supplementary Figure S1 | SSC surface markers expression in SSC cultured on laminin, DTM hydrogel (10 mg/ml) and Matrigel.** **(A)** FACS analysis of the expression of SSC surface proteins. The red histograms represent the cell count for the specific antibody, and the back histograms represent the fluorescence of the negative control. **(B)** Statistical analysis of the results of flow cytometry.


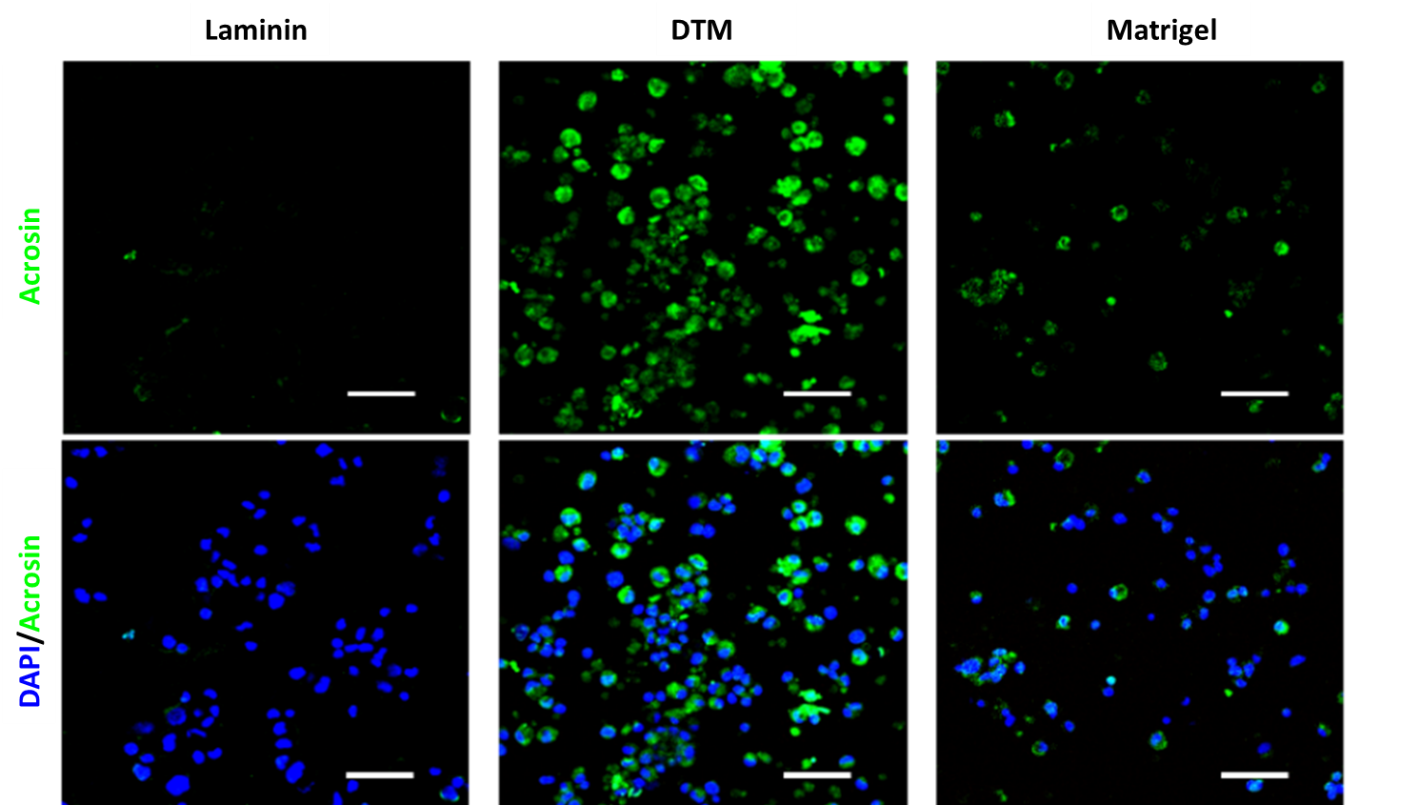


**Supplementary Figure S2 | Representative images of Acrosin immunofluorescence in the differentiated cells from SSCs**. The scale bars represent 50 μm.
